# Artificial intelligence and machine learning approaches for patient safety in complex surgery: a review

**DOI:** 10.1186/s13037-025-00458-8

**Published:** 2025-11-25

**Authors:** Mohamed Mustaf Ahmed, Zhinya Kawa Othman, Uthman Okikiola Adebayo, Omar Kasimieh, Olalekan John Okesanya, Shuaibu Saidu Musa, Francesco Branda, Victor C. Cañezo Jr., Edgar G. Cue, Don Eliseo Lucero Prisno III

**Affiliations:** 1 Faculty of Medicine and Health Sciences, SIMAD University, Mogadishu, Somalia; 2 Department of Pharmacy, Kurdistan Technical Institute, Sulaymaniyah, Kurdistan Region Iraq; 3 Department of Medical Laboratory Science, Neuropsychiatric Hospital, Aro, Abeokuta, Nigeria; 4 University of the East Ramon Magsaysay Memorial Medical Center, Quezon City, Philippines; 5 Department of Public Health and Maritime Transport, University of Thessaly, Volos, Greece; 6 School of Global Health, Faculty of Medicine, Chulalongkorn University, Bangkok, Thailand; 7 Unit of Medical Statistics and Molecular Epidemiology, Campus Bio- Medico University of Rome, Rome, Italy; 8 Office of the University President, Biliran Province State University, Naval, Leyte, Philippines; 9 Office of the University President, Mountain Province State University, Bontoc, Mountain Province Philippines; 10 Department of Global Health and Development, London School of Hygiene and Tropical Medicine, London, UK; 11 Office for Research, Extension and Innovations, Bukidnon State University, Malaybalay City, Bukidnon, Philippines; 12 Research Office, Palompon Institute of Technology, Palompon, Leyte, Philippines

**Keywords:** Artificial intelligence, Machine learning, Surgery, Patient safety, Complex surgery

## Abstract

Artificial intelligence (AI) and machine learning (ML) are increasingly being used in surgical care; however, their real-world impact on patient safety is not well established. This narrative review searched PubMed, Scopus, and Google Scholar for English-language studies published from January 1, 2015, to April 30, 2025, that evaluated AI and ML applications in complex surgery and reported quantitative patient safety outcomes. Eligible included studies were published between 2016 and 2025. In total, 21 studies were synthesized across the preoperative, intraoperative, and postoperative phases of the study. Preoperatively, ML models consistently outperformed traditional risk scores in identifying high-risk patients and anticipating technical difficulties. Intraoperatively, AI-enabled decision support reduced hypotension exposure in a randomized trial, and computer vision systems supported the safety-critical step verification and instrument tracking. Postoperatively, multimodal approaches combining electronic records, imaging, and smartphone wound photographs predicted complications, such as surgical site infection, and facilitated discharge planning. Emerging evidence from ambulatory surgery, imaging-guided triage, and specialty domains, alongside qualitative studies on workforce readiness, highlights implementation opportunities and human factor requirements. Most evidence is retrospective, single-center, or prototype stage with limited external validation and uncertain generalizability across settings, including low- and middle-income countries. Priorities include multicenter prospective trials, standardized outcomes and reporting, continuous bias and model drift monitoring, robust data infrastructure, and equity-focused implementation to translate algorithmic performance into fewer complications, deaths, and costs.

## Introduction

Globally, an estimated 310 million major surgical procedures are performed every year [1]; however, up to 25% f inpatients experience at least one postoperative complication, and the crude perioperative mortality ranges from 0.5% to 5% [2]. These hazards translate into approximately 4.2 million deaths within 30 days of surgery [3] and help drive the >3 million annual deaths attributed to unsafe care, with one in ten patients harmed overall [4]. Historic safety initiatives prove that risk is modifiable: the World Health Organization (WHO) Surgical Safety Checklist reduced inpatient complications from 11% to 7% and mortality from 1.5% to 0.8% across eight countries [5, 6]. Artificial intelligence (AI) and machine learning (ML) are increasingly being integrated into complex surgical care. Recent work frames this as a shift from retrospective audit to proactive, machine-learning–enabled anticipation and prevention of harm across the perioperative continuum [7].

Preoperatively, an extreme-gradient-boosting model trained on 66 846 elective cases achieved an AUROC of 0.95 for predicting in-hospital mortality while providing patient-specific explanations [8], and systematic reviews showed similar gains across bariatric and cardiac cohorts [9, 10]. This trajectory extends beyond inpatient pathways: in ambulatory surgery, a recent review catalogs current AI applications and practical challenges around workflow integration, governance, and evaluation [11]. Intraoperatively, computer-vision platforms quantify surgeon performance with pixel-level fidelity; the latest dual-model framework reached an intersection-over-union of 0.93 and Dice 0.87 for vessel segmentation while distinguishing expert from novice microsurgeons [12], and automated video analytics consistently correlate technical metrics with adverse event rates [13].

Human-factor considerations remain central, and qualitative research with robotic surgical nurses describes evolving roles, trust, and training needs as AI tools enter the operating room [14]. Deep neural networks ingesting high-resolution physiological streams during 56 242 operations lifted discrimination for sepsis (AUROC 0.80 vs. 0.78) and venous thrombo-embolism (0.74 vs. 0.71) over random forests [15]. Postoperatively, a multimodal neural network predicted surgical site infection within 48 h with clinician-level accuracy (AUROC 0.777 vs. 0.762) while cutting staff review time by 80% (51.5 h to 9.1 h) [16], and large-scale early warning systems now report AUROC >0.90 for major adverse events six hours before onset [17]. Specialty-focused overviews (e.g., benign prostate surgery) similarly report emerging utility alongside persistent gaps in validation and standardization that must be closed to translate performance into outcomes [18].

There is a paucity of literature on whether these predominantly retrospective, high-income country models generalize to low- and middle-income settings, remain robust to data drift, preserve equity across age, sex, and comorbidity strata, or translate AUROC gains into fewer deaths, complications, and costs in real-world workflows [19]. Bridging these evidence gaps is essential for aligning AI innovation with the Global Patient Safety Action Plan 2021–2030, enabling regulators to assign appropriate risk classes and guiding hospitals to strategically invest in scarce digital health budgets to maximize safety. This narrative review aims to critically synthesize current evidence on AI-enabled interventions for patient safety across the pre-, intra-, and postoperative continuum in complex surgery, identify persistent methodological and policy deficits that hinder real-world impact, and articulate a translational research agenda capable of turning algorithmic promises into measurable, equitable improvements in surgical outcomes.

## Methods

We performed a narrative literature review to identify peer-reviewed studies that evaluated artificial intelligence (AI) technologies for patient safety outcomes before, during, and after complex surgical procedures. PubMed, Scopus, and Google Scholar were searched for articles published between January 1, 2015, and April 30, 2025. Search strings combined AI terms (“artificial intelligence,” “machine learning,” “deep learning,” “computer vision,” “natural language processing,” “robotic surgery,” “decision support”) with patient safety outcome terms (“complication,” “adverse event,” “morbidity,” “mortality,” “error,” “infection,” “readmission”) and stage descriptors (“preoperative,” “intraoperative,” “postoperative”) using Boolean operators AND and OR; Medical Subject Headings (MeSH) were applied, where available. Eligible sources were original investigations (randomized trials, cohort, case–control, cross-sectional, and diagnostic test accuracy studies), narrative reviews, and systematic reviews that (i) examined any AI application in a surgical setting, (ii) reported quantitative patient safety outcomes, and (iii) were published in English. Grey literature and non-peer-reviewed abstracts were excluded. Two reviewers (M.M.A. and O. K.) screened the titles, abstracts, and full texts; disagreements were resolved by consensus, and the reference lists of the included papers were hand-searched for additional titles. Data were extracted into a standardized matrix capturing the author, year, study design, surgical stage, specialty, key performance metrics, reported clinical impact, and stated challenges or limitations, and presented in three data extraction tables (preoperative, intraoperative, and postoperative). The findings were narratively synthesized by surgical stage and AI application to highlight the observed effectiveness and barriers to implementation.

## Results

### Preoperative AI applications: risk stratification and surgical planning

Twenty-one eligible studies were included. The prespecified search window was from January 1, 2015, to April 30, 2025. No eligible studies were published in 2015. The included studies were published between 2016 and 2025, with majority published after 2020. Most published research is retrospective or cross-sectional, whereas only a small number of studies are randomized or prospective [20–22]. Preoperative studies have mainly focused on risk stratification and complexity forecasting, especially in emergency general surgery, colorectal, and hepatobiliary procedures [23–27]. During surgery, the main aim was hypotension prevention and safety confirmation, as well as instrument tracking, real-time histopathology, and robotic autonomy [21, 28–31]. Postoperative performance is mainly focused on discharge planning, remote wound surveillance, and complication prediction, such as respiratory failure, surgical site infection, and cerebral ischemia [16, 22, 32–34]. These points make it clear that governance frameworks and multicenter trials are necessary for the safe, effective, and widespread application of AI technology.

AI tools applied before incision reliably outperform traditional scores in identifying high-risk patients and anticipating technical difficulties (Table 1). Hashimoto et al. framed the field, highlighting privacy, bias and workflow challenges while underscoring the surgeon’s role [35]. Bertsimas et al. then introduced the gradient-boosted Predictive OpTimal Trees in Emergency Surgery Risk (POTTER) calculator: across 51,457 emergency operations, the model achieved a c-statistic of 0.916 for 30-day mortality, which was well above American Society of Anesthesiologists (ASA) class and American College of Surgeons National Surgical Quality Improvement Program (ACS NSQIP) [23]. It also generated patient-specific complication probabilities via an intuitive dashboard [23]. External validation in 4,171 emergency laparotomy and general surgery cases confirmed excellent calibration and an area under the receiver operating characteristic curve (AUROC) of 0.93 [20].

Large multi-institution datasets have enabled the development of automated early warning systems. Mahajan et al. deployed a neural network at 20 US hospitals that flagged 13% of 1.25 million surgical patients [24]. By doing so, 52% of ensuing deaths were captured, allowing targeted prophylactic measures and multidisciplinary involvement [24]. Xue et al. fused 87 pre-operative and 94 intra-operative variables from 42,202 mixed procedures and integrated models incorporating both phases achieved AUROCs of 0.83–0.90 for major complications which allowed for 4–7% points better than pre-operative data alone [25]. Procedure-specific planning models are emerging. Yu et al. trained an interpretable XGBoost algorithm on pelvic magnetic resonance imaging (MRI) measurements and routine lab tests to predict technically difficult laparoscopic total mesolectal excision, yielding an AUROC of 0.86 with 84% accuracy nd 92% specificiy in the test set [26]. While external validation is pending, the model illustrates how AI can alert surgeons to challenging anatomy and prompt referral or a modified operative strategy. A systematic review of 43 AI-based preoperative decision support tools across eight specialties concluded that most improved discrimination versus legacy tools but suffered from heterogeneous reporting and scarce benchmark datasets, limiting cross-study comparison [27].

### Intraoperative AI applications: real-time decision support, computer vision and autonomy

Within the operating room, AI interventions mitigated hemodynamic instability and flagged technical lapses (Table 2). In the HYPE randomized clinical trial (*n* = 60 elective noncardiac surgeries), an ML-derived early warning system reduced the depth and duration of mean arterial pressure < 65 mmHg fourfold compared with standard care (median time-weighted MAP 0.10 vs. 0.44 mmHg · min; *P* = 0.001) [21]. Computer vision (CV) platforms can address two recurring safety hazards. Deol et al. achieved 99.4% pecision in real-time detection and counting of 11 laparoscopic instruments which operates as an automated safeguard against retained items [29]. Mascagni et al. trained a deep network to verify attainment of the critical view of safety (CVS) during laparoscopic cholecystectomy; balanced accuracy was 72%, enabing an on-screen prompt when dissection remained unsafe [36].

Rapid intraoperative pathology was enhanced by Hollon et al. whose convolutional neural network classified brain tumor specimens from stimulated Raman histology within 2.5 min and with 94.6% accuracy, matching frozen section diagnostics [30]. At the frontier of autonomy, the Smart-Tissue Autonomous Robot executed ex vivo porcine bowel anastomoses with suture precision and leak pressures comparable to those of expert surgeons [31]. For higher-level decision support, Shin et al. applied unsupervised clustering to 570 adult spinal deformity cases, uncovering four phenotypes with distinct complication profiles and opening the door to real-time, data-driven intraoperative risk stratification [28]. Nevertheless, a systematic review of AI in robot-assisted surgery found scant prospective evidence that current systems translate to improved patient outcomes, underscoring the need for multicenter trials and human–AI team workflows [37].

### Postoperative AI applications: complication prediction, remote monitoring and discharge optimization

After surgery, the AI models accurately predicted adverse events and streamlined recovery pathways (Table 3). Safavi et al. created a ward-based neural network that forecasts next-day discharges (AUROC 0.84), where prospective use in 605 patients freed 128 bed-days without increasing readmissions [22]. Hadaya et al. mined more than 1 million emergency general surgery cases to build an XGBoost model for predicting acute respiratory failure, with an AUROC of 0.90 [34]. There were patients flagged as high risk who had greater than 10-fold higher mortality and markedly longer stays [33].

**Fig. 1:**
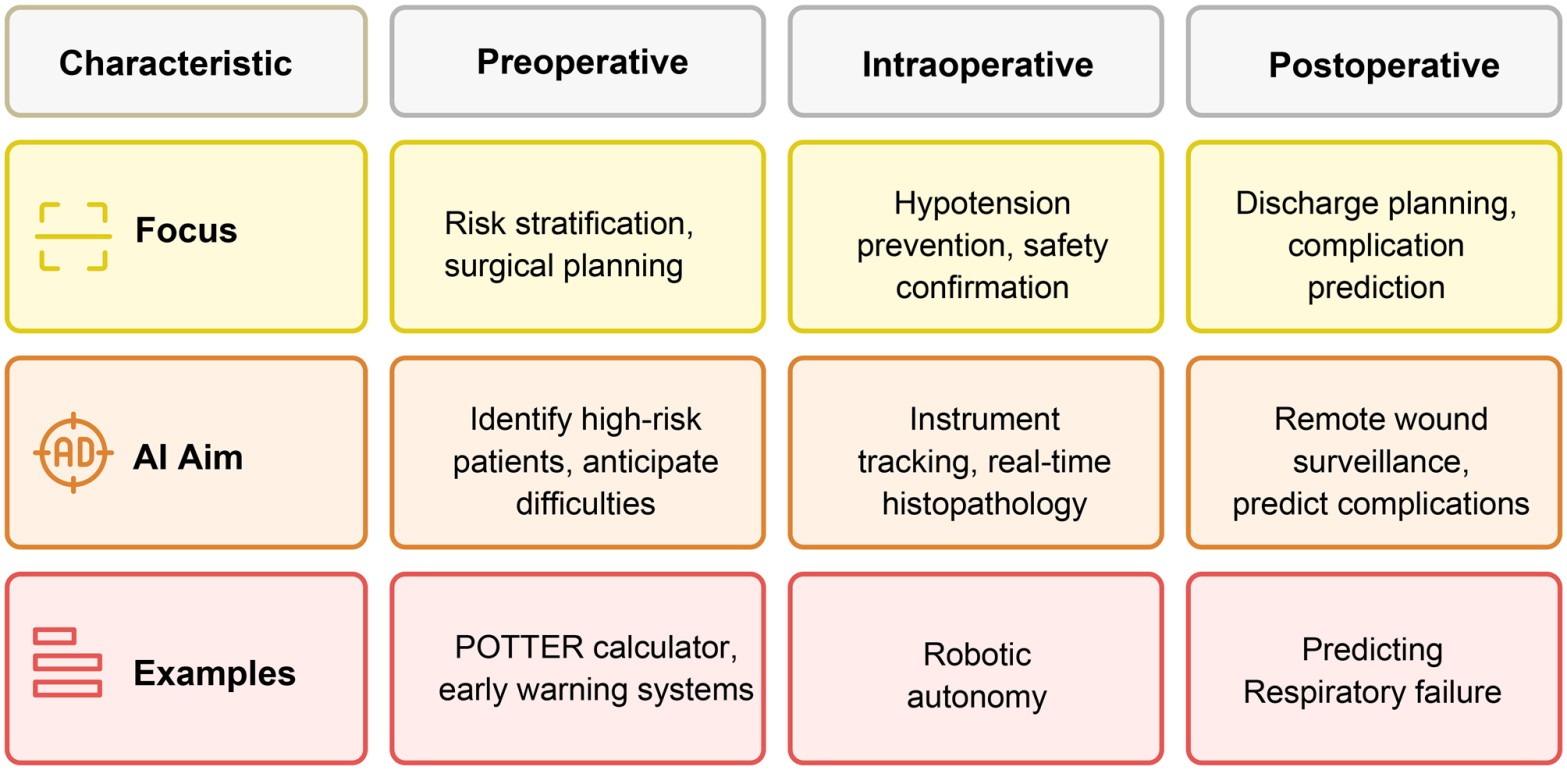
AI applications in surgery. POTTER = Predictive OpTimal Trees in Emergency Surgery Risk

Remote patient-generated data streams are now actionable. McLean et al. combined smartphone wound photographs with symptom surveys that employed a multimodal deep learning model detected surgical site infection 48 h before clinical diagnosis (AUROC 0.76) while cutting manual surveillance workload by 80% [16]. In neurologic critical care, Hu et al. integrated early computed tomography (CT) and physiological data to forecast delayed cerebral ischemia after subarachnoid hemorrhage (AUROC 0.88), outperforming conventional scores [33]. Oncology applications are also advancing; a scoping review by Ravenel et al. indexed machine learning nomograms that stratify digestive surgery patients’ long-term outcomes and guide adjuvant therapy decisions [32]. AI systems have improved preoperative risk stratification, curtailed intraoperative hypotension and technical errors, and accurately predicted diverse postoperative complications (Fig. 1). The strongest evidence is a randomized hemodynamic trial that demonstrated a fourfold reduction in hypotension, whereas large multisite early warning models have already reshaped perioperative workflows. However, most tools remain single-center, retrospective, or in the prototype stage. Rigorous multicenter trials and standardized reporting are essential before routine clinical use.

**Table 1:** AI impact on patient safety – Preoperative stage (risk stratification & planning)

Study (Author, year)	Reference	Design	Specialty	Key findings / Outcomes	Challenges / notes
Hashimoto et al. 2018	[35]	Narrative review	General	Outlined AI subfields; emphasized surgeon role in safe AI use	Data privacy, algorithm bias, and integration into clinical workflow identified as major challenges.
Bertsimas et al. 2018	[23]	Retrospective cohort	General (Emergency)	Provided interactive, nonlinear risk assessment for mortality and complications.	Prospective validation in diverse settings needed
El Hechi et al. 2021	[20]	Diagnostic test accuracy study	General (Emergency)	Machine learning model displayed high accuracy in mortality	Emphasized need for integration into clinical workflow (e.g. preop counseling) and caution in different hospital settings due to data variations.
Mahajan et al. 2023	[24]	Diagnostic test accuracy study	All (Mixed surgeries)	Automated preop risk model accurately identified patients at high risk of 30-day mortality complications.	High performance in large dataset; requires ongoing monitoring for bias.
Xue et al. 2021	[25]	Retrospective cohort study	All (Mixed surgeries)	Combined pre/intraoperative data to predict post operative complications	Large single-center study; performance may vary in external settings.
Kapoor et al. 2025	[38]	Diagnostic test accuracy study	General Surgery (HPB)	ML model predicted surgical difficulty with approximately 90% accuracy	Requires broader validation
Yu et al. 2024	[26]	Analytical cross-sectional study	Colorectal (Oncology)	Developed a model to predict complexity of rectal surgery (LaTME).	Pending external validation of model
Choudhury & Asan, 2020	[27]	Systematic Review	Multiple specialties	AI-based decision support systems enhancing patient risk stratification preoperatively	Noted heterogeneity in reporting and lack of standard benchmarks.

Note: AI = Artificial Intelligence; ML = Machine Learning; HPB = Hepatopancreatobiliary; LaTME = Laparoscopic Total Mesorectal Excision; preop = preoperative; intraoperative = during the operation; postoperative = after the operation

**Table 2:** AI impact on patient safety – Intraoperative stage (decision support, automation & error reduction)

Study (Author, year)	Reference	Design	Specialty	Key findings / outcomes	Challenges / notes
Wijnberge et al. 2020	[21]	Randomized control trial	Anesthesiology	AI early-warning system predicted hypotension before it occurred	This was a single-center preliminary trial. Larger multicenter trials needed to confirm outcome benefits.
Deol et al. 2024	[29]	Analytical Cross-sectional study	Urology (robotic)	Developed a real-time computer vision model to count surgical tools, aiming to prevent retained items.	Study not yet in human surgeries.
Mascagni et al. 2022	[36]	Analytical Cross-sectional study	General Surgery (HPB)	AI model automatically assessed CVS criteria achievement during gallbladder surgery.	Moderate accuracy – unable to catch all nuances
Hollon et al. 2020	[30]	Diagnostic test accuracy study	Neurosurgery	Used a deep neural network with stimulated Raman histology to diagnose brain tumor specimens intraoperatively	Implemented in specialized centers with SRH equipment; generalization to all pathology types needs work
Moglia et al. 2021	[37]	Systematic review	Multiple (Robotic)	Surveyed AI in robotic surgery: autonomous suturing, gesture recognition, and skill assessment algorithms.	Lack clinical validation
Shin et al. 2019	[28]	Case Control Study	Orthopedic (Spine)	Applied models to adult spinal deformity cases to group patients by key features and predict outcomes	Provided a new risk classification beyond conventional surgeon intuition
Shademan et al. 2016	[31]	Diagnostic test accuracy study	General Surgery	STAR successfully performed intestinal anastomoses in ex vivo porcine models with minimal human intervention.	Conducted in laboratory conditions on animal tissue, not clinical use.

Note: AI = Artificial Intelligence; HPB = Hepatopancreatobiliary; CVS = Critical View of Safety; SRH = Stimulated Raman Histology; STAR = Smart Tissue Autonomous Robot

**Table 3:** AI impact on patient safety – Postoperative stage (monitoring, complications prediction & response)

Study (Author, Year)	Reference	Design	Specialty	Key Findings / Outcomes	Challenges / Notes
Safavi et al. 2019	[22]	Cohort study	General Surgery	A neural network predicted which post-surgical inpatients would be discharged within 24 h	Model was single center; performance may vary elsewhere
Ravenel et al. 2023	[32]	Retrospective study	General Surgery	Applied machine learning to colorectal cancer patients with bilobar liver metastases to identify prognostic factors and develop a nomogram predicting overall survival.	Many models lack external validation and are retrospective. In addition, Heterogeneity in endpoints and sample sizes makes generalizability difficult.
McLean et al. 2025	[16]	Diagnostic test accuracy study	Colorectal (GI surgery)	Combined patient-reported symptoms and wound to predict surgical site infection within 48 h.	Developed in a controlled study; real-world adherence to remote monitoring can affect performance.
Hu et al. 2022	[33]	Diagnostic test accuracy study	Neurosurgery	Applied machine learning to early clinical and imaging data after subarachnoid hemorrhage to predict delayed cerebral ischemia with greater accuracy than traditional approaches.	Highly specific context (neurocritical care); requires continuous multimodal data inputs
Hadaya et al. 2022	[34]	Analytical cross-sectional study	Emergency surgery	Developed a predictive model for post-op respiratory failure in emergency general surgery patients	Focused on only one complication (ARF) using registry data.
Shickel et al. 2023	[15]	Analytical cross-sectional study	All (Mixed inpatient surgery)	Applied a deep neural network on surgeries to predict nine common 30-day complications	Complex model requiring extensive EHR integration

Note: GI = Gastrointestinal; post-op = postoperative; ARF = Acute Respiratory Failure; EHR = Electronic Health Record

## Discussion

Artificial intelligence tools are now being designed for every phase of complex surgery; however, most published evidence remains in the early stages and is from single centers. A recurring pattern in the literature is that algorithms achieve impressive performance metrics in silico or in narrowly defined settings but only sporadically demonstrate measurable gains in real-world patient safety. Recent studies have continuously demonstrated that ML models, especially those employing gradient boosting and random forests, are superior to conventional risk scores, such as ASA and NSQIP, in forecasting surgical mortality and complications. For instance, a large experiment using random forest classifiers on more than 49,000 surgical patients produced an AUC of 0. 921 for predicting in-hospital mortality, which was higher than the AUCs of both logistic regression and the ASA Physical Status Classification. Integrating preoperative and intraoperative data into deep neural networks further enhanced predictive accuracy, with AUCs reaching 0. 924, and demonstrated that patient risk changes over time and can be tracked longitudinally. At a systems level, big-data surgical registries and trauma databases are being coupled with ML to operationalize continuous safety surveillance and decision support at scale, reinforcing the need for standardized data pipelines and governance [39].

These ML-based methods exclusively use structured electronic medical record data, allowing for completely automated risk forecasting without the need for clinician-derived scores and providing more personalized and dynamic risk assessments than traditional methods. However, before widespread implementation can take place, reliable external validation and smooth integration into clinical processes are still necessary [40]. With randomized trials showing a fourfold reduction in hypotension exposure during elective noncardiac surgery [25, 41], AI-derived early warning systems, such as the one evaluated in this review, have demonstrated a significant reduction in both the depth and duration of intraoperative hypotension compared with standard care. Thermoregulatory safety is another intraoperative target: explainable ML has shown promise for predicting hypothermia risk during laparoscopic surgery, broadening the scope of AI-assisted prevention beyond hemodynamics [42].

Similar benefits have been observed in cardiac surgery, where machine learning-based hypotension prediction tools have reduced the severity and duration of hypotension by more than 60% without raising blood pressure or requiring more medication [43]. Additional randomized trials have confirmed that these systems not only lower the occurrence of hypotensive episodes and the amount of time spent in hypotension, but also lower biomarkers of organ injury and oxidative stress, indicating the potential for improved patient outcomes [44, 45]. A Systematic review and meta-analysis further supported that AI-driven predictive analytics, especially the Hypotension Prediction Index (HPI), consistently outperformed standard monitoring by reducing intraoperative hypotension and demonstrating strong predictive accuracy [46]. Nevertheless, most data originate from single-center or controlled environments, and the long-term effects on patient outcomes and cost-effectiveness remain unclear, underscoring the need for multicenter trials and more extensive validation before widespread clinical use [47].

Multimodal deep learning models that combine wound images with patient-reported symptoms have demonstrated significant potential for the early detection of SSI and for reducing the workload of manual monitoring. Compared to clinician triage, the model, like the one created in this review, identified SSIs up to 48 h prior to clinical diagnosis and had similar diagnostic accuracy while lowering the staff time needed for monitoring by 80% [16]. Broader research, which highlights the necessity of external validation and meticulous integration into clinical workflows, supports these findings by demonstrating that automated, image-based, and multimodal machine learning methods can match or even outperform clinicians in detecting SSI [48]. Additionally, animal model studies have shown that multimodal sensors and machine learning systems can predict SSIs up to 48 h in advance, further corroborating the possibility of early, automated detection [49].

Pathology-focused AI also contributes to safety by standardizing detection and grading, as shown in validated work on prostatectomy specimens [50]. Across surgical subspecialties, focused overviews in spine surgery and benign prostate surgery echo our conclusions that growing predictive performance is tempered by heterogeneous reporting, limited external validation, and a need for prospective, outcome-oriented trials before routine use [51]. Preoperatively, non-invasive ML models that integrate ultrasonography are being leveraged to guide selective lymphadenectomy in thoracic esophageal cancer, illustrating how AI can reduce overtreatment and perioperative risk when thoughtfully integrated into decision pathways [52]. Nonetheless, obstacles persist, such as ensuring the capture of high-quality data, keeping patients engaged, and incorporating these technologies into current treatment pathways to optimize their efficacy and scalability for regular practice [48].

### Strengths and limitations

One of the major strengths of this review is its thorough coverage of the pre-, intra-, and postoperative continuum, which integrates data from various surgical disciplines and study methodologies. The review includes both technical performance measures and early indicators of real-world impact by incorporating excellent prospective and randomized trials. For example, the incorporation of massive multi-institutional datasets, such as Mahajan et al. ‘s neural network analysis of more than a million patients, improves the generalizability of the results and facilitates comparisons across various clinical contexts. However, the nature of the available data restricted the review. Most of the included studies were retrospective or single-center and were frequently conducted in high-income environments, which raises questions about external validity and relevance to low- and middle-income nations. Some promising AI tools have been validated only in regulated laboratory settings, whereas others have not been validated externally. Direct comparison between studies is also difficult because of heterogeneity in outcome definitions, input variables, and performance reporting, as emphasized in systematic reviews.

### Implications for policy, practice and future research

**Fig. 2:**
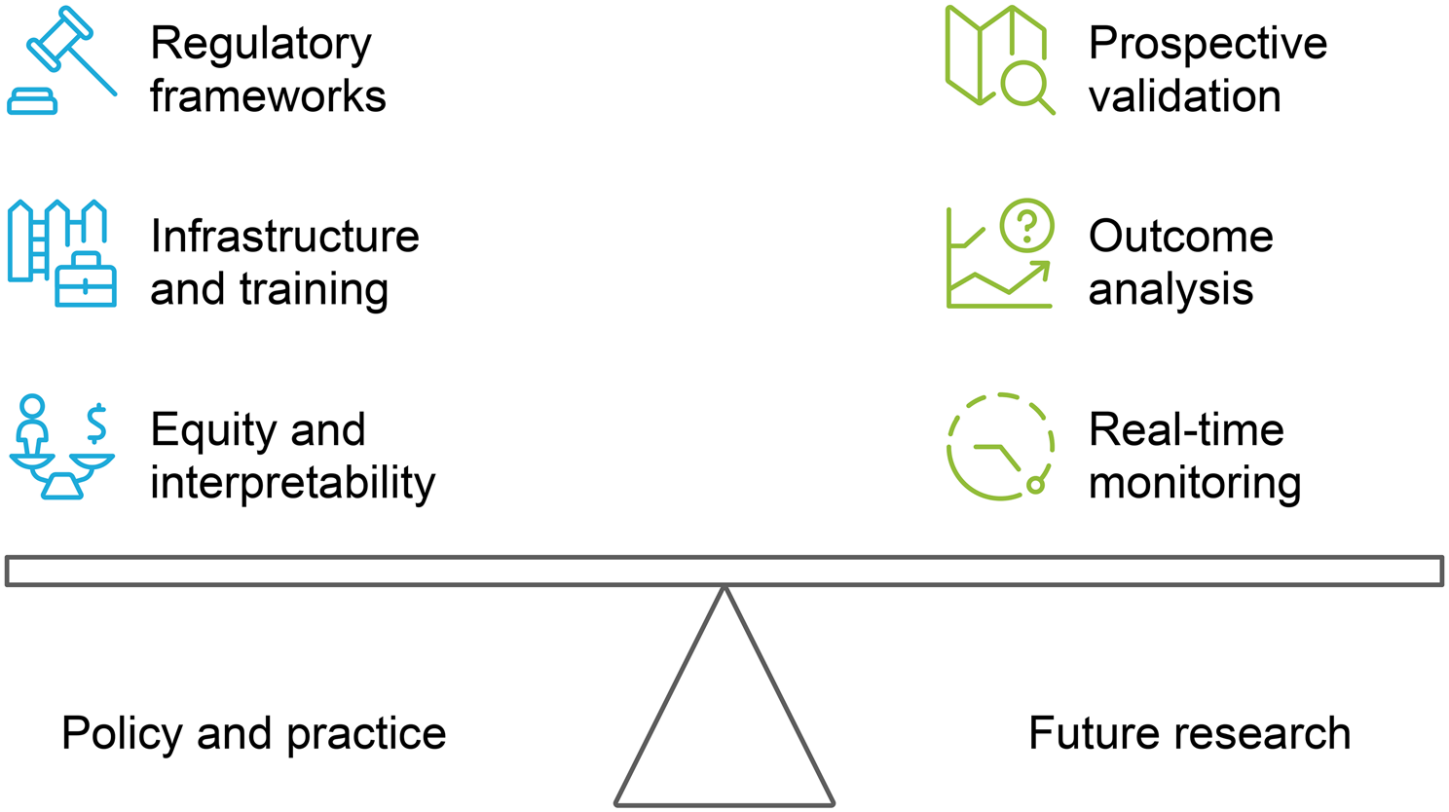
Balancing policy, practice, and research in AI integration

AI technologies have the potential to improve patient safety throughout the surgical process; however, turning these gains into regular treatments necessitates specific policies, practical approaches, and research methodologies (Fig. 2).

From a policy standpoint, regulatory frameworks should be changed to tackle the specific challenges of AI, such as transparency, bias reduction, and adaptive approval procedures that enable iterative model improvement while protecting patient safety. To ensure the smooth integration of AI tools into perioperative decision-making without adding to the cognitive load of healthcare professionals, hospitals and healthcare systems should invest in digital infrastructure, interdisciplinary training, and workflow redesigns. Implementation should be guided by an equity-focused design with proactive auditing of model performance across various care settings and patient subgroups. To encourage clinician confidence and continuous adoption, AI outputs must be interpretable, timely, and consistent with the current clinical procedures.

To determine generalizability and cost-effectiveness, future studies should prioritize the prospective multicenter validation of promising tools, especially in diverse geographic and resource contexts. In addition to predictive accuracy, research should also analyze how AI-driven insights impact actual patient outcomes and investigate ways to integrate real-time performance monitoring into deployed systems. By concentrating innovation on these priorities, AI may develop from experimental prototypes into trustworthy copilots that demonstrably improve surgical safety across the globe.

## Conclusion

AI and ML are now involved in every phase of complex surgery, from preoperative risk stratification to intraoperative prevention and postoperative surveillance. Signals of benefit include stronger preoperative discrimination than legacy scores, reduced intraoperative hypotension in randomized evaluations, and earlier detection of postoperative complications with workload reduction. Translation to routine safety gains remains limited by retrospective designs, single-center development, heterogeneous reporting, and scarce external validation. Human factors readiness and governance are equally pivotal, as shown by qualitative work in robotic surgery and the need for explainability, training pathways, and workflow-compatible interfaces. System-level enablers, such as data registries, standardized pipelines, and ongoing performance auditing, are necessary to sustain safe deployment and detect bias and drift. Future work should emphasize multicenter prospective trials, outcome-oriented implementation studies across diverse settings, and cost-effectiveness analyses. With these elements in place, AI and ML can evolve from promising prototypes to equitable and auditable tools that can measurably reduce harm and improve surgical outcomes.

## Data Availability

Not applicable; no new datasets were generated or analyzed.
